# A Case of Prostato-Cystitis with Complication

**Published:** 1900-09

**Authors:** J. B. H. Day

**Affiliations:** Social Circle, Ga.


### A CASE OF PROSTATO-CYSTITIS WITH COMPLICATION.*

By J. B. H. DAY, M.D.,

Social Circle, Ga.

As denoted by my caption, it is not of prostato-cystitis in general that I propose to speak, but of a particular case. Moreover, it is not so much to the disease itself as to the embarrassment of certain complications that I shall invite your attention.

In the year 1891 the patient to whose case I shall refer, having had already a good deal of trouble in micturating, was taken with obstinate and severe retention of urine resulting from obstruction caused by a much enlarged prostate gland. This condition was, after a time, relieved, but was followed by a cystitis which subsided only to give place to a chronic vesicular catarrh, and a permanent inability to void urine without the use of a catheter.

The hypertrophied gland continued to enlarge up to the summer of 1898, when it reached the size of a large orange, and a portion of it protruded some distance into the bladder; thus necessitating the use of an extra long specially curved metallic instrument.

About this time an alarming hematuria set in accompanied by intense pain and inflamation of both the gland and the bladder. Of course the first indication was to evacuate the bladder, but upon the introduction of the catheter it was found that its contents were not of such a character as to flow. A large coagulum had formed of sufficient firmness to be distinctly felt through the abdominal wall.

A recurved instrument was at hand, but could it have been introduced would not have been of any more utility than the simple

*Read before the Medical Association of Georgia, at Atlanta, April, 1900.

one. The clot became larger, the distention and pain increased; then there was considerable pyrexia followed by pronounced toxemia which soon became profound, the patient becoming unconscious both to surroundings and to pain. It was fast becoming a serious question as to what should be done to remove the decomposing contents of that cavity, for it was quite evident that without its accomplishment an early death was imminent. So precarious had become the situation that even suprapubic cystotomy suggested itself as a last resort.

However, the patient's age was over seventy, which fact, taken together with his general condition, precluded the advisability of such a method of procedure. Therefore the question resolved itself into whatever method might be successful *per urethram.*

The only *available* instruments at hand were the catheter, already *in situ,* and an ordinary household syringe. After a few moments of meditation the syringe was attached to the catheter, the valves being so placed as to produce suction *from* the bladder. This plan, which at first was thought to be a happy inspiration, a little later seemed destined to end in woful disappointment; for when the bulb was compressed it failed to expand, so firm had become the clot within the diseased organ.

Not yet completely baffled, another idea soon offered itself. The rubber tube of syringe was firmly grasped near its attachment to the catheter between the thumb and fingers of one hand and tightly compressed, while that hand was brought forward along the tube up to the first valve. Without yet releasing the grasp of this hand the other hand was passed over the same portion of tube in like manner. After a number of repetitions of this series of actions the bulb began to expand and was not long in being filled. By continued manipulation in this way the flow was established and the bladder was at last emptied of a large quantity of putrescent blood and ammoniacal urine which emitted a most powerful, sickening and offensive odor.

The syringe was then readjusted and the bladder thoroughly irrigated with a warm solution of permanganate of potassium of the strength of about 1 to 2000. The irrigation was kept up at intervals in connection with appropriate general treatment, for several weeks ; at the end of which time the patient had regained his previous state of health. Nevertheless, he has subsequently suffered from two severe attacks of orchitis, first of one testicle and then of the other, both attacks resulting in suppuration of such violence and extent as to well-nigh bring about spontaneous castration. Under antiseptic treatment, with the free use of peroxide of hydrogen, these complications terminated favorably.

Those who believe in the efficacy of castration in cases of this character would expect some diminution in the size of the prostate gland. I cannot state that the gland is smaller than it was two years ago, though it may not have become at all increased.

In conclusion I will say that the general health and condition of the patient is now pretty fair for one of his age. His improvement and approach to recovery has been all that could be expected in cases of this class. In common with the rest of the profession I have to deplore the fact that we have not more effective means of relieving or reducing hypertrophy of the prostate gland.

---

INTESTINAL obstruction treated by the hypodermic injection of large doses of atropine is reported by Dr. Batsch.* In each of the three cases in which it was used, purgative medication had been ineffectual, the abdomen was tender and much distended, and stercoraceous vomiting had occurred. The dose of atropine administered was 0.005 gm. (1-12 grn.). A single injection sufficed for the relief of one patient, while another required two before the lost function was restored with simultaneous disappearance of the distressing and alarming symptoms. In the third case the treatment failed for the reason, as demonstrated at the subsequent laparotomy, that the obstruction was due to a band of fibrous tissue, beneath which the intestine had been caught and strangulated. Although this condition had lasted some time, as proven by the marked constriction rings at the ends of the incarcerated portion of the bowel, and although that portion was purple when first seen, severance of the fibrous band was followed almost immediately by restoration of color and function and the prompt recovery of the patient without a single untoward symptom. To this happy result the doctor believes that the atropine largely contributed.

* Münch. med. Woch., XLVII., p. 931.

